# Molecular identification of *Acetobacter pasteurianus *and *Lactobacillus fermentum*, contaminating an ethanol industry in the state of Pernambuco

**DOI:** 10.1186/1753-6561-8-S4-P200

**Published:** 2014-10-01

**Authors:** Mariana Sampaio, Glícia Calazans, Márcia Silva

**Affiliations:** 1 Universidade Federal de Pernambuco, Recife, PE, Brasil

## Introduction

*Acetobacter pasteurianus *is among the major bacteria responsible for acetic fermentation and gives the characteristic taste of vinegar, can be found as a contaminant of wine production industry. In industrial fermentation is frequent contamination by lactic acid bacteria such as *Lactobacillus fermentum *that competes with the yeast for nutrients and inhibiting their growth, slow the fermentation and impair the production of ethanol[1]. (AIM) The objective of this study is the molecular identification of bacteria isolated as contaminants of an ethanol industry in the state of Pernambuco, by sequencing the 16S ribosomal gene (16S rDNA).

## Methods

The samples were cultured in a sucrose syrup at 35°C for 24 hours and subjected to DNA isolation using the protocol of Sambrook et al (1989) [2] by extraction with phenol / chloroform and precipitation with isopropanol. The PCR reaction was performed using 16S rDNA primers then analyzed by agarose gel electrophoresis observed in UV transilluminator and revealed. The amplified product of the 16S rDNA gene was purified, sequenced and the data were aligned with the tool BLASTn (Basic Lenth Alignamment Search Tool) algorithm enabling the identification by comparison to other sequences deposited in the GenBank database from NCBI (National Center for Biotechnology Information).

## Results and conclusions

The PCR products of the gene 16S rDNA of the bacteria had a molecular weight of approximately 1500 base pairs [3]. Thus, the samples showed to be able to step sequencing and soon after had their sequences compared in the database and showed high similarity (99 %) with *Acetobacter pasteurianus *and *Lactobacillus fermentum *. One of the bacteria showed up as producer of biopolymer, *L. fermentum *, this drew attention by quantity and rheological properties of a polymer produced molasses medium. The molecular technique used revealed a high degree of conservation of the 16S rDNA gene and comparison of the sequence obtained in this study with sequences deposited in GenBank database resulted in strains of known species showing similarity with the sequences under study.
